# Nonlinear Feature Extraction Through Manifold Learning in an Electronic Tongue Classification Task

**DOI:** 10.3390/s20174834

**Published:** 2020-08-27

**Authors:** Jersson X. Leon-Medina, Maribel Anaya, Francesc Pozo, Diego Tibaduiza

**Affiliations:** 1 Departamento de Ingeniería Mecánica y Mecatrónica, Universidad Nacional de Colombia, Cra 45 No. 26-85, Bogotá 111321, Colombia; jxleonm@unal.edu.co; 2 MEM (Modelling-Electronics and Monitoring Research Group), Faculty of Electronics Engineering, Universidad Santo Tomás, Bogotá 110231, Colombia; maribelanaya@usantotomas.edu.co; 3 Control, Modeling, Identification and Applications (CoDAlab), Department of Mathematics, Escola d’Enginyeria de Barcelona Est (EEBE), Universitat Politècnica de Catalunya (UPC), Campus Diagonal-Besòs (CDB), Eduard Maristany, 16, 08019 Barcelona, Spain; 4 Departamento de Ingeniería Eléctrica y Electrónica, Universidad Nacional de Colombia, Cra 45 No. 26-85, Bogotá 111321, Colombia; dtibaduizab@unal.edu.co

**Keywords:** manifold learning, feature extraction, classification, electronic tongue, machine learning, t-SNE, LTSA, isomap, locally linear embedding

## Abstract

A nonlinear feature extraction-based approach using manifold learning algorithms is developed in order to improve the classification accuracy in an electronic tongue sensor array. The developed signal processing methodology is composed of four stages: data unfolding, scaling, feature extraction, and classification. This study aims to compare seven manifold learning algorithms: Isomap, Laplacian Eigenmaps, Locally Linear Embedding (LLE), modified LLE, Hessian LLE, Local Tangent Space Alignment (LTSA), and *t*-Distributed Stochastic Neighbor Embedding (*t*-SNE) to find the best classification accuracy in a multifrequency large-amplitude pulse voltammetry electronic tongue. A sensitivity study of the parameters of each manifold learning algorithm is also included. A data set of seven different aqueous matrices is used to validate the proposed data processing methodology. A leave-one-out cross validation was employed in 63 samples. The best accuracy (96.83%) was obtained when the methodology uses Mean-Centered Group Scaling (MCGS) for data normalization, the *t*-SNE algorithm for feature extraction, and *k*-nearest neighbors (*k*NN) as classifier.

## 1. Introduction

Sensor arrays that are composed of electrochemical sensors can be used to discriminate different types of aqueous matrices, preserve flavor, detect anomalies, or quantify any analyte within an aqueous matrix [1]. The system known as the electronic tongue is composed of an array of non-selective sensors made of different materials that have the cross sensitivity property with which independent signals are captured by each sensor [2]. The process is managed by an electronic data acquisition component that controls the test carried out with the sensors, which in the case of electrochemicals measures, for example, can be voltammetric or potentiometric [3]. Finally, the data are sent to the pattern recognition system responsible for processing signals through multivariate data analysis and machine learning algorithms. Signal processing in electronic tongues sensor arrays aims to solve a classification or regression problem via machine learning algorithms [4].

In 2007, Tian et al. [5] developed the multifrequency large amplitude pulse voltammetry (MLAPV) method for electronic tongues. This method is a combination of waveforms of LAPV with different frequencies. The MLAPV electronic tongue has better discrimination ability thanks to the combination of several sensors of different materials and specific frequency segments in the voltammetry excitation signal. The MLAPV electronic tongue has been successfully used in applications of classification of aqueous matrices such as yogurt [6], tea [7], rice wine [8], black tea [9], monofloral honeys [10], and waters [11]. Furthermore, the MLAPV electronic tongue has demonstrated its correct application in the classification of different types of substances into data sets of seven [12,13] and thirteen [14] different aqueous matrices.

It is necessary to consider the feature extraction in the process, as most studies in classification makes use of pattern recognition [15]. Feature selection and feature extraction are two different steps to treat features on patter recognition tasks, the first attempts to select relevant features of the raw data and eliminate irrelevant features. Furthermore, feature extraction methods are dimensionality reduction techniques that transform the features of the raw data while preserving the content of global information [16]. Different methods to perform the dimensionality reduction process include: (a) Feature Extraction from Original Response Curves, (b) Feature Extraction from Curve Fitting Parameters, (c) Feature Extraction from Transform Domain, and (d) Feature Extraction from Phase Space [17].

This work shows a pattern recognition methodology for MLAPV electronic tongue classification. One of the stages in this methodology is dimensionality reduction, which is conducted using manifold learning, when considering that the signals obtained by each sensor in the MLAPV electronic tongue have a particular manifold [18]. Manifold learning searches the intrinsic low-dimensional embedding structures within high-dimensional data [19]. It has demonstrated its effectiveness in applications, such as hyperspectral data [20], financial markets [19], high-dimensional datasets [21], and structural health monitoring (SHM) for damage classification [22,23]. There are two types of manifold learning methods: local and global. The global approach tries to preserve geometry at all scales. Conversely, the local approach pursue to map close points in the manifold to close points in the low dimensional representation. Local approaches can become more effective and provide useful results in a wider range of manifolds by improving its representational capacity [24].

Dimensionality reduction should not only be used for visualization or as pre-processing on very high dimensional data, but also as a general pre-processing technique on numerical data to raise the classification accuracy [25]. In recent years, some works related to feature extraction algorithms and their use in classification tasks in sensor arrays have been developed. For instance, Zhang and Tian in 2014 [26] developed the kernel discriminant analysis method to perform classification in an electronic nose. Other studies related to the use of manifold learning algorithms in electronic nose-type sensor arrays include [27,28,29]. Among the books with relevant topics from the perspective of signal processing, manifold learning, and machine learning strategies in electronic nose sensor arrays is [30]. In 2019, Zhu et al. [31] used principal component analysis (PCA), linear discriminant analysis (LDA), Kernel PCA (KPCA), and Laplacian Eigenmaps in an electronic nose automatic system for predicting the freshness of crabs. In 2020, Leon-Medina et al. [32] developed a machine learning classification methodology based on nonlinear feature extraction algorithms applied to solve odor recognition problems in electronic noses. The methodology was based on the combination of some dimensionality reduction algorithms and a k-nearest neighbors (*k*NN) classifier to perform a holdout cross validation in a dataset of six gases and a total of 3600 measurements. A study on tea quality gradation with the application of manifold learning algorithms to extract effective features in a potentiometric electronic tongue was developed in [33]. The study found that better results were obtained by kernel LDA and kernel Locality preserving projections (KLPP). In [34], different machine learning algorithms are compared in order to determine the best behavior in an electronic tongue. The authors found that random forests was the best classifier. Finally, the work of Gutierrez et al. in 2013 [35] emphasizes the features extracted from the signal shape in a cyclic voltammetric electronic tongue.

Different works related to signal processing of a MLAPV electronic tongue have been developed in recent years. In 2018, Zhang et al. [14] developed a MLAPV electronic tongue that uses feature selection through a filter-based approach to select a feature vector, enter a subspace learning method, called local discriminant preservation projection (LDPP), and ultimately trained a kernel extreme learning machine that serves as classification algorithm. In their study, 5-fold cross validation was executed in an imbalanced data set of 13 different aqueous matrices and 114 measurements, reaching a classification accuracy of 98.22%. An active feature selection (ASF) strategy was developed in 2018 by Liu et al. [13]. The ASF strategy is based on a discrete wavelet transform (DWT) to select features from a MLAPV electronic tongue. Four machine learning classifiers were compared: (*k*NN), Support Vector Machines (SVM) with linear and radial basis function kernels, and Random Forest. The best classification accuracy with a hold-out cross validation procedure was achieved by the combination of the ASF-DWT method with *k*NN, reaching a value of 84.13% with standard deviation of ±0.0125. An alternative feature extraction method named feature specificity enhancement (FSE) for dimensionality reduction in a MLAPV electronic tongue sensor array was also proposed by Liu et al. in 2020 [12]. The authors combined an extreme learning machine classifier with the FSE method to obtain 95.24% of classification accuracy in a hold-out cross validation methodology with a data set of 7 different aqueous matrices. Table 1 shows a summary of the works related to signal processing in MLAPV electronic tongues. The reader can refer to [3] for an extensive review in signal processing methodologies in electronic tongues.

As a contribution, this work aims to present an artificial taste recognition methodology for signal processing in MLAPV electronic tongues, based on the use of manifold learning algorithms in the dimensionality reduction stage. The methodology explores new ways to carry out multivariate data analysis and properly perform the pattern recognition stage showing the high relevance of the algorithms’ subject for interpreting data from electronic tongues. The current manuscript is different from the previous published works, since it uses the Mean Centered group scaling method as the pre-processing stage to normalize the data that are obtained by sensors of different materials in the electronic tongue. Seven different manifold learning algorithms are compared: Isomap, Locally Linear Embedding (LLE), Laplacian Eigenmaps, modified LLE, Hessian LLE, Local Tangent Space Alignment (LTSA), and *t* -Distributed Stochastic Neighbor Embedding *t*-SNE. Besides, we also compare the performance of five different supervised machine learning classifiers: KNN, SVM, multi layer perceptron artificial neural network (MLP ANN), adaptive boosting (Adaboost), and Gaussian process classifier. It evaluates the influence of parameter variation and target dimensions in the classification accuracy on a data set of seven different aqueous matrices. This data set is characterized by being multiclass, balanced and a small sample. Furthermore, to the best of our knowledge, *t*-SNE is the first attempt of data processing in electronic tongues, therefore, this works aims to give a variation to the classic PCA method, which is widely used by the chemometric community and provides a new way of processing data in electronic tongues.

The structure of the paper is as follows. Section 2 reports on the theoretical background of each step of the developed methodology: scaling, feature extraction, classification, and validation. Next, the Section 3 describes the data set of seven aqueous matrices used to validate the current methodology. The artificial taste recognition methodology is explained in detail in Section 4. Subsequently, Section 5 shows the results and discussion obtained by applying the methodology in a dataset of 7 different aqueous matrices. Section 6 outlines the main conclusion of this research.

## 2. Theoretical Background

Signal processing in an electronic tongue sensor array is a data fusion process performed for artificial taste recognition. Data come from several sensors of different materials; consequently, as in the human tongue, there are millions of taste buds. Cross sensitivity is used to obtain signals from each sensor in different aqueous matrices and, thus, be able to identify them. In this research, manifold learning algorithms for nonlinear feature extraction are used to obtain a group of features at the entrance to different supervised machine learning classifiers, following some important concepts and methods are briefly described.

### 2.1. Electronic Tongue

**Figure 1:**
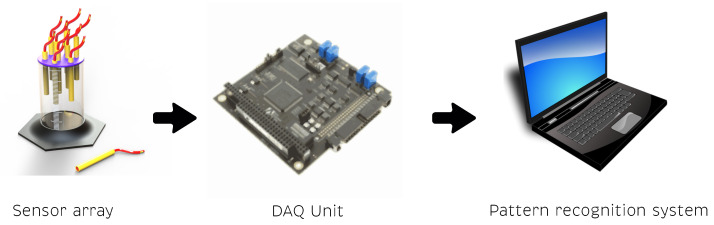
Electronic tongue components.

Electronic tongues are devices composed of three main parts. First, an electrochemical cell composed of the sensor array that allows the interaction with the analytes. Second, an electronic data acquisition unit that controls the current, voltage, and frequency in the electrochemical measures. This, through a central processing unit and D/A and A/D converters, allows for communication with a computer. The measurements are recorded and processed by an external computer. As third component, a multivariate data analysis is performed by a pattern recognition system, thus determining qualitative or quantitative results. Figure 1 illustrates the main parts of an electronic tongue sensor array.

### 2.2. Data Unfolding

The response measures that are obtained by an MLAPV electronic tongue are referred to as discretized currents in time. Thereby, a measure is obtained by each one of the electrodes that composed the electronic tongue. Following the formulation proposed by Pozo et al. [39], the discretized measurements of the sensor can be arranged to form a matrix, as follows [1]:

(1) $$\begin{array}{c} X=\left(\begin{array}{cccc} x_{11} & x_{12} & \cdots & x_{1K}\\ \vdots & \vdots & \ddots & \vdots \\ x_{i1} & x_{i2} & \cdots & x_{iK}\\ \vdots & \vdots & \ddots & \vdots \\ x_{I1} & x_{I2} & \cdots & x_{IK} \end{array}\right)\in M_{I\times K}\left(R\right). \end{array}$$

This matrix X$\in M_{I\times K}\left(R\right)$, where $M_{I\times K}\left(R\right)$ is the vector space of I×K matrices over R, which contains information from I∈N experimental trials and K∈N time instants.

When considering that, in the case of the electronic tongue, an array of sensors is used, J∈N is the number of sensors (working electrodes) at each experiment, and there is a number *J* of the aforementioned matrix (Equation (1)). Thus, the resulting three-way data matrix with I×J×K has to be unfolded to obtain a two-way matrix following an unfolding procedure through the E-type method [40]. In the E-type method, for each sensor, the matrices presented in Equation (1) are concatenated to create a larger matrix $X\in M_{\left(I)\times (J\cdot K\right)}\left(R\right)$, as follows [39]:

(2) $$\begin{array}{c} X=\left(\begin{array}{cccccccccccccc} x_{11}^{1} & x_{12}^{1} & \cdots & x_{1K}^{1} & | & x_{11}^{2} & \cdots & x_{1K}^{2} & | & \cdots & | & x_{11}^{J} & \cdots & x_{1K}^{J}\\ \vdots & \vdots & \ddots & \vdots & | & \vdots & \ddots & \vdots & | & \ddots & | & \vdots & \ddots & \vdots \\ x_{i1}^{1} & x_{i2}^{1} & \cdots & x_{iK}^{1} & | & x_{i1}^{2} & \cdots & x_{iK}^{2} & | & \cdots & | & x_{i1}^{J} & \cdots & x_{iK}^{J}\\ \vdots & \vdots & \ddots & \vdots & | & \vdots & \ddots & \vdots & | & \ddots & | & \vdots & \ddots & \vdots \\ x_{I1}^{1} & x_{I2}^{1} & \cdots & x_{IK}^{1} & | & x_{I1}^{2} & \cdots & x_{IK}^{2} & | & \cdots & | & x_{I1}^{J} & \cdots & x_{IK}^{J} \end{array}\right). \end{array}$$

### 2.3. Mean-Centered Group Scaling

The data collected by the MLAPV electronic tongue are arranged as a matrix *X* in Equation (2); these data come from several sensors, and the magnitudes measured by these sensors may have different scales [41]. Thus, the data must be rescaled applying a pre-processing stage. One method for scaling the data is the mean-centered group scaling (MCGS) [42]. Group scaling is frequently used when the data have several blocks of equal variables. In this case, the blocks are the number of sensors *J* and each block comprises variables in some given units of measure, but different sensors use different units [1]. In MCGS, the mean of all measurements of the sensor in the same column is considered in the normalization, as follows [39]:

(3) $$\begin{array}{cc} \mu^{N} & =\frac{1}{IK}\sum_{i=1}^{I}\sum_{k=1}^{K}x_{ik}^{N},\;N=1,2,\ldots ,J \end{array}$$

(4) $$\begin{array}{cc} \sigma^{N} & =\sqrt{\frac{1}{IK}\sum_{i=1}^{I}\sum_{k=1}^{K}{\left(x_{ik}^{N}-\mu^{N}\right)}^{2}},\;N=1,2,\ldots ,J \end{array}$$

where $\mu^{N}$ and $\sigma^{N}$ are the mean and the standard deviation of all the elements in matrix $X^{N}$, respectively. More precisely, $\mu^{N}$ and $\sigma^{N}$ are the mean and the standard deviation of all the measurements of sensor *J*, respectively.

In MCGS, the mean of all measurements of the sensor in the same column is considered in the normalization. More precisely, we define the following:

(5) $$\begin{array}{c} \mu_{k}^{N}=\frac{1}{I}\sum_{i=1}^{I}x_{ik}^{N},\;\;k=1,\ldots ,K,\;N=1,2,\ldots ,J \end{array}$$

where $\mu_{k}^{N}$ is the arithmetic mean of the measurements located at the same column, which is, the mean of the *I* measurements of sensor *J* in matrix $X^{N}$. Therefore, the elements $x_{ik}^{N}$ of matrix *X* would be scaled—using MCGS––to create a new matrix $\overset{⌣}{\;X}$ = *X_MCGS_* = ( ${\overset{⌣}{\;x}}_{ik}^{N}$ ) as

(6) $${\begin{array}{c} \overset{⌣}{\;x} \end{array}}_{ik}^{N}:=\frac{x_{ik}^{N}-\mu_{k}^{N}}{\sigma^{N}},\;\;i=1,\ldots ,I,\;\;\;\;k=1,\ldots ,K,\;\;N=1,2,\ldots ,J$$

### 2.4. Dimensionality Reduction

Owing to the large number of measurement points obtained in each experiment, unfolding is carried out in such a way that the data of each sensor are ordered one next to the other. There is a matrix of size (I)×(J·K), where *I* is the number of experiments, *J* the number of sensors, and *K* is the number of measurement points per experiment. However, the (J·K) quantity is characterized by a high dimensionality. Therefore, a feature extraction step is run to reduce dimensionality and create a feature vector of a considerably smaller size, which contains relevant information for each class, in order to facilitate the classifying for machine learning algorithms, such as *k*NN. Different dimensionality reduction techniques are reported in the literature to eliminate irrelevant and redundant features. The selection of an appropriate feature extraction method can help enhance the processing speed and reduce the time and effort required to extract valuable information [43].

### 2.5. Manifold Learning

In this work, manifold learning [44] is used as a dimensionality reduction technique. Seven different nonlinear manifold learning algorithms—Isomap, LLE, Laplacian Eigenmaps, modified LLE, Hessian LLE, LTSA, and *t*-SNE—were compared to identify the best one in terms of classification accuracy. In the following sections, these algorithms are briefly described.

#### 2.5.1. Isomap

Isomap [45] is a dimensionality reduction method that aims to preserve geodesic distances. This distance between two vertices is the length in terms of the number of edges of the shortest path between the vertices. This method is a variant of the multidimensional scaling algorithm in which the Euclidean distances are substituted by geodesic distances. After finding the geodesic distances, the next step is to run multidimensional scaling, performing the eigende composition of the Gram matrix and selecting the λ most important eigenvectors to represent the low-dimensional space [46].

#### 2.5.2. Locally Linear Embedding

The LLE [47] is a manifold learning algorithm where each sample point can be linearly represented by its closest neighbors [48]. LLE eliminates the need to estimate pairwise distances between widely separated data points and, in this method, the sampled data resides locally linear patch of the manifold. The LLE method consist on three stages (1) select neighbors, (2) reconstruct with linear weights, and, finally, (3) map to embedded coordinates. For further details, the reader is referred to the original paper of Roweis and Saul [47].

#### 2.5.3. Laplacian Eigenmaps

Laplacian Eigenmaps [49] is a local nonlinear dimensionality reduction algorithm that uses the Laplacian of the graph concept for finally solving a sparse eigenvalue problem. In Laplacian Eigenmaps, local properties are based on pairwise distances between close neighbors [50]. Laplacian Eigenmaps is composed and three stages (1) constructing the graph, next (2) choosing the weights and finally (3) Eigenmaps. For further details, the reader is referred to the original work of Belkin and Niyogi [49].

#### 2.5.4. Modified LLE

The modified LLE [51] is based on multiple linearly independent local weight vectors for each neighborhood. Modified LLE shows the existence of linearly independent weight vectors that are approximately optimal. The local geometric structure that is determined by multiple weight vectors is much stable and improves the original LLE algorithm [51]. For further details, the reader is referred to the original paper of Zhang and Wang [51].

#### 2.5.5. Hessian LLE

Hessian LLE [52] is a local dimensionality reduction technique. The Hessian matrix represents information on the curviness of the high-dimensional data manifold. An Eigenanalysis of the Hessian matrix is performed in order to find the low-dimensional data representation that minimizes the curviness of the manifold. In Hessian LLE, a constraint that the low-dimensional data representation is locally isometric is imposed. Hessian LLE replaces the manifold Laplacian of the original LLE algorithm by a new manifold Hessian [53].

#### 2.5.6. Local Tangent Space Alignment (LTSA)

LTSA [54] represents the local geometry of the manifold using tangent spaces learned by fitting an affine subspace in a neighborhood of each data point. Those tangent spaces are aligned to give the internal global coordinates of the data points concerning to the underlying manifold by way of a partial Eigendecomposition of the neighborhood connection matrix [54]. The procedure of LTSA algorithm is as follows: identify neighbors, obtain tangent coordinates, develop Hessian estimator, develop quadratic form, find approximate null space, perform an Eigenanalysis, and identify the dimensional subspace corresponding to the smallest eigenvalues. Finally, find basis for null space.

#### 2.5.7. *t*-Distributed Stochastic Neighbor Embedding (*t*-SNE)

*t*-SNE [55] reduces the tendency to crowd points in the center of the distribution. *t*-SNE is an improved version of the SNE [56] algorithm, the latter is hampered by a cost function that is difficult to optimize. There are two main stages in the *t*-SNE method. (1) A probability distribution is predictable among the pairs of high-dimensional data points, so that similar objects are assigned a high probability of being selected and dissimilar points are assigned small probability of being chosen. (2) *t*-SNE designates a uniform probability distribution model in the low-dimensional map by minimizing the Kullback—Leibler divergence [57]. The authors have successfully used the *t*-SNE algorithm in other research, such as [42,58]. For a detailed explanation, the authors suggest the reading of the *t*-SNE original work [55].

### 2.6. Supervised Machine Learning Classifiers

Five different supervised machine learning algorithms were compared in the classification stage, among them were: KNN, SVM, MLP ANN, Adaboost, and Gaussian process classifier. Because of these classifiers being well known, the authors suggest the reading of the following works, for more details [59,60,61].

The parameters used in this study for each one of the classifiers were for *k*NN the number of neighbors was set to two and an Euclidean distance was used. For SVM, a cubic kernel function, box constraint level equal to one, and one vs one multiclass method were used. For the MLP ANN alpha was equal to one and a maximum of 1000 iterations was set. For Adaboost, the number of estimators was equal to 50 and we use a learning rate of 1. Finally, for the Gaussian process classifier, a radial basis function kernel was used.

### 2.7. Leave-One-Out Cross Validation

The leave-one-out cross validation (LOOCV) technique was used to verify the correct behavior of the developed artificial taste recognition methodology. LOOCV is used due to the few experiments conducted. In this sense, the use of LOOCV avoids the possible overfitting in the classification model. In the literature, some works adopt leave-one-out cross validation in order to evaluate the performance of a classification algorithm when the number of instances in a data set or that for a class value is small [62].

### 2.8. Performance Measure

In this work, the classification performance that is used to compare and evaluate the behavior of the developed methodology is the classification accuracy, defined as the ratio of correct classifications over the total number of samples. This accuracy is shown in the following equation:

(7) $$\begin{array}{c} \mathrm{accuracy}=\frac{\mathrm{TP}+\mathrm{TN}}{\mathrm{TP}+\mathrm{TN}+\mathrm{FP}+\mathrm{FN}} \end{array}$$

where TP are true positive, TN are true negative, FP are false positive, and FN are false negative values in the confusion matrix obtained after perform LOOCV.

## 3. Dataset of a MLAPV Electronic Tongue

In 2018, Liu et al. [13] developed a MLAPV electronic tongue composed of an auxiliary electrode sensor of pillar platinum, a reference electrode sensor of Ag/AgCl, and six working electrodes of different materials, such as gold, platinum, palladium, titanium, tungsten, and silver. In the experiments, the fourth electrode was broken. For this reason, the data obtained by the titanium electrode were not considered in data analysis.

For each drink, three different concentrations of the original solution were mixed with distilled water. These three concentrations were 14%, 25%, and 100%, named as low, medium, and high concentrations. Three replicates were made for each of the concentrations for a total of nine samples per aqueous matrix [13]. Therefore, the data set is composed of a total of 63 measurements. Tests were conducted on seven different aqueous matrices: red wine, white spirit, beer, black tea, oolong tea, maofeng tea, and pu’er tea. The data set used to validate the developed artificial taste recognition methodology is shown in Table 2.

## 4. Artificial Taste Recognition Methodology

The pattern recognition methodology developed in this study consists first in having the raw data obtained by the multi-frequency and large amplitude pulse voltammetry tests with the electronic tongue of five different materials. These data correspond to a size of 2050 measurement points per sensor. Second, the data arrangement and unfolding is performed. In this way, different data obtained by each sensor are ordered one after another toward the right, forming a feature vector per each measure of 10,250 measurement points. As previously explained, the data set used to validate the current signal processing methodology of MLAPV electronic tongues consists in measuring seven different aqueous matrices, with nine samples per class for a total of 63 measurements. Therefore, the final unfolded matrix has a size of 63 × 10,250. Subsequently, the third stage is data normalization, where the data obtained from the different sensors are scaled through the MCGS method for taking into account differences in the measures obtained by each material in the aqueous matrix. Subsequently, in the fourth stage, a dimensionality reduction procedure is achieved through manifold learning algorithms. In this study, a comparative study of seven different algorithms is conducted to determine the best one in terms of classification accuracy when varying the number of target dimensions and the proper parameters of each manifold learning algorithm. These algorithms perform the feature extraction stage by minimizing intra-classes distances and maximizing inter-classes distances between each class of aqueous matrix.

**Figure 2:**
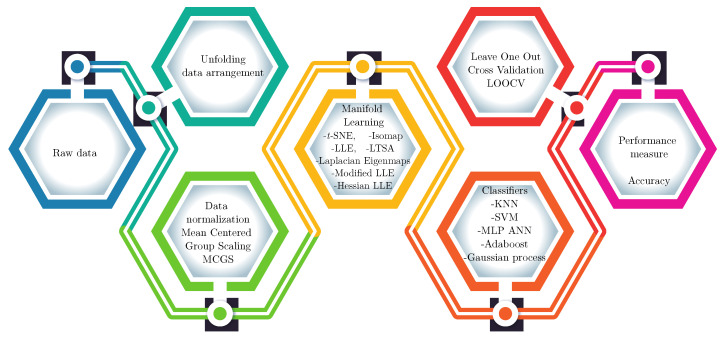
Manifold learning based artificial taste recognition methodology.

The dimensionality reduction stage allows for to obtain a feature vector of low size, thus facilitating the work done by the machine learning classification algorithm in stage five. In this case, five different supervised machine learning algorithms were compared. The next stage in the methodology is process validation with the LOOCV method, in which one sample is trained and evaluated with the others. The LOOCV method is used for the few samples available for each class. After the cross validation process, a confusion matrix is obtained. Finally, in the last stage, the accuracy classification is calculated from the confusion matrix obtained. The six stages that compose the developed artificial taste recognition methodology previously are shown in Figure 2.

The fine tuning of the different parameters of each algorithm is an important process to achieve a high classification accuracy. Therefore, the first parameter to configure is the number of groups in the MCGS method. In this case, the electronic tongue is composed of five electrodes, so the number of groups is set to five. The next parameter to configure is the k number of neighbors belonging to the manifold learning algorithm used. We searched in a range from seven to 63 neighbors, in order to find the best accuracy calculated through the KNN classifier and LOOCV. Finally, the target dimension to embed the scaled data is selected according to the high accuracy found in a range from two to 17 dimensions.

## 5. Results and Discussion

### 5.1. MCGS Scaling

**Figure 3:**
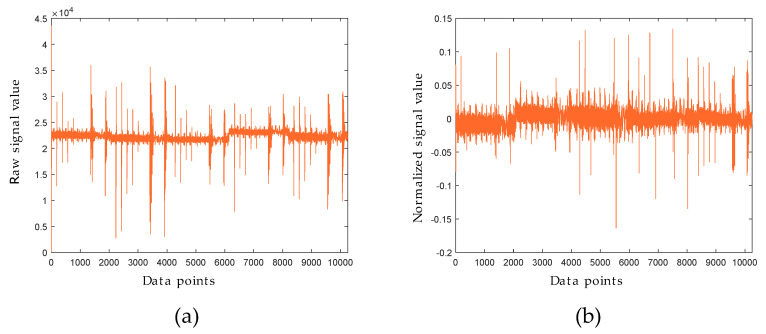
(**a**) Original signal versus (**b**) normalized signal by mean-centered group scaling (MCGS) method.

The excitation signal of a MLAPV electronic tongue includes several frequency segments in one time cycle in order to stimulate different transient pulse-like responses. Better discrimination ability can be achieved by the combination of working electrodes with specific frequency segments. Unfolded raw data from the five working electrodes are shown in Figure 3a. The different response peaks can be observed in Figure 3. The multiple frequency pulse signal used in the MLAPV electronic tongue is composed by three different frequencies, 0.2 Hz, 1 Hz, and 2 Hz, as well as five different pulse amplitudes 1 V, 0.8 V, 0.6 V, 0.4 V, and 0.2 V. Figure 3b shows the scaling values after applying the MCGS method.

### 5.2. Manifold Learning, Dimensionality Reduction and Classification

**Figure 4:**
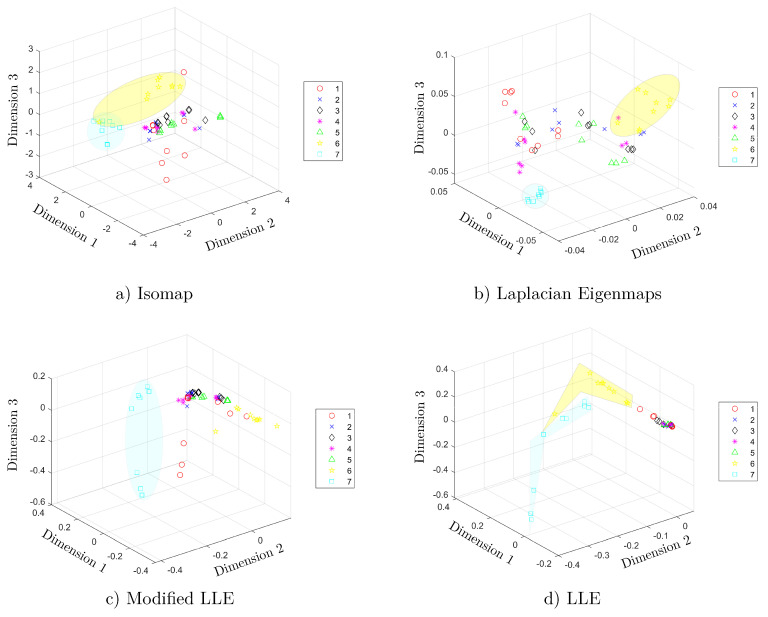
Three dimensional scatter plots after applying (**a**) Isomap; (**b**) Laplacian Eigenmaps; (**c**) Modified Locally Linear Embedding (LLE); and (**d**) LLE. The numbers in the legend refers to the seven classes of aqueous matrices, as follows: red wine (1) white spirit (2), beer (3), black tea (4), oolong tea (5), maofeng tea (6), and pu′er tea (7).

In the study conducted by Zhang in 2018 [14], a filter and the selection of 30 features per electrode were used to form a 150th dimension feature vector. In this study, algorithms of manifold learning were used for performing a feature extraction stage. In this sense, the features extracted properly belong to the manifold and do not correspond at any time to features of the waveform. A comparative study is conducted with seven manifold learning algorithms: Isomap, Laplacian Eigenmaps, modified LLE, Hessian LLE, LTSA, LLE, and *t*-SNE. The number of target dimensions that form the feature vector at the input of each supervised machine learning classifier has a minimum of two dimensions and a maximum of seventeen. The scatter plots of the manifold learning algorithms are depicted in Figure 4, showing the first three dimensions of each manifold learning algorithm.

Figure 4a shows the three-dimensional scatter plot of the Isomap algorithm. The index of each class corresponds to the list of the seven aqueous matrices listed in Table 1. Figure 4a shows that the first five aqueous matrices are mixed, and it is not possible to differentiate them. Conversely, there is a clear separation of the sixth and seventh aqueous matrices, corresponding to maofeng tea and pu’er tea, respectively. A similar situation occurs in the modified LLE (Figure 4c) and LLE (Figure 4d) methods. In Figure 4b, the Laplacian Eigenmaps method shows results where the classes are mixed together; therefore, a supervised classification algorithm is needed and, in this case, it allows for the correct classifying of different classes in the dataset.

The next step in the artificial taste recognition methodology is to execute a classification stage once the feature vector is formed, utilizing the named manifold learning methods. In this case, a *k*NN classifier is used with Euclidean distance, and a hold out cross validation is performed. Consequently, a confusion matrix is obtained in every classification process.

**Figure 5:**
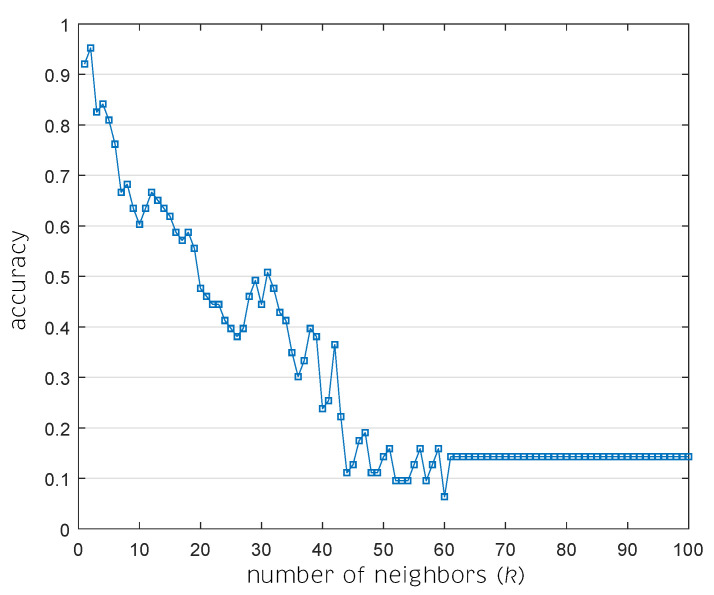
Accuracy behavior due to the variation of neighbors number in *k*NN classifier algorithm.

A study on the variation of the parameter of *k* neighbors in the *k*NN is performed. As shown in Figure 5, the best result achieved was an average accuracy of 96.83%, when k=2 neighbors was used. The behavior of this *k* parameter of the classifiers has a tendency to decrease accuracy as *k* increases.

**Figure 6:**
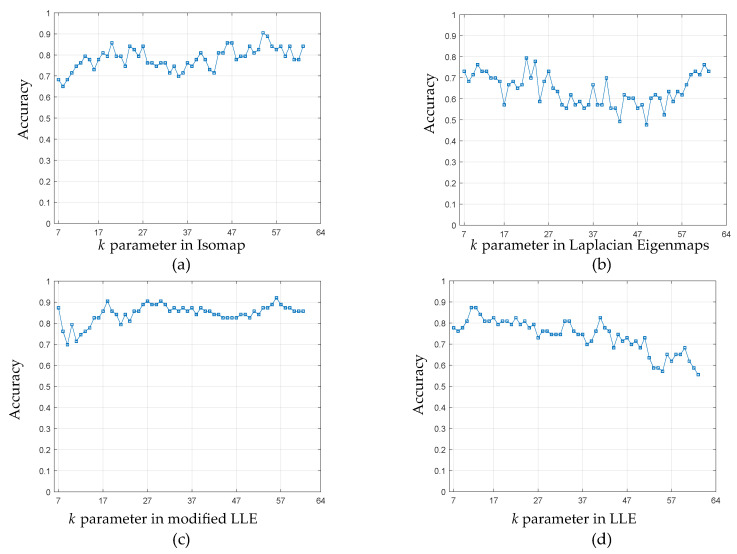
Accuracy sensitivity of *k* parameter variation in (**a**) Isomap; (**b**) Laplacian Eigenmaps; (**c**) Modified LLE; and, (**d**) LLE.

A hyper parameter must be tuned in each manifold learning algorithm to determine the best values—in this case, according to the behavior in the accuracy. The parameter variation in each manifold learning method is shown in Figure 6. The Isomap, Laplacian Eigenmaps, modified LLE, Hessian LLE, LTSA, and LLE have the same *k* hyperparameter in common due to the nature of the methods, where the local properties are based on pairwise distances between near neighbors. The value of *k* varies between 7 and 63, because 7 is the minimum number of samples per class and 63 is the total number of samples in the classes.

Consequently, in Figure 6a the best value of *k* for the Isomap algorithm was k=54 with an accuracy of 90.48%. Isomap has a tendency to increase the precision as *k* increases, in the Figure 6b, k=22 for Laplacian Eigenmaps and a maximum accuracy of 80.16%. In general, the behavior is oscillating and varies from a minimum accuracy of 48% for Laplacian Eigenmaps. In Figure 6c, the best value of *k* was 56 for modified LLE with an accuracy of 92.06%. Subsequently, it slightly descends and remains constant. Finally, in the Figure 6d the best value for *k* was k=11 for LLE, with an accuracy of 88.00%. Otherwise, in the case of LLE as *k* increases, the accuracy decreases.

**Figure 7:**
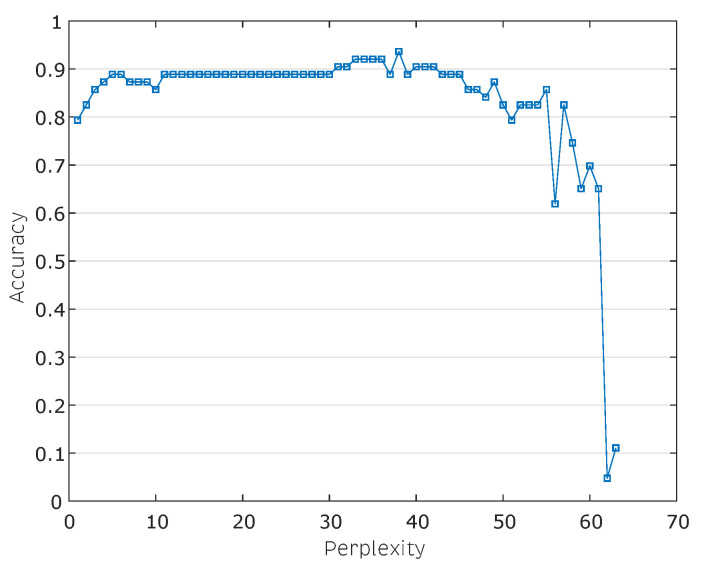
Effect of the perplexity parameter variation belonging to the algorithm *t*-Stochastic Neighbor Embedding (SNE) on the classification accuracy.

The effect of the perplexity variation in the *t*-SNE algorithm and its influence on the classification accuracy is shown in Figure 7. The perplexity can be interpreted as a smooth measure of the effective number of neighbors. The performance of *t*-SNE is fairly robust to changes in the perplexity, and the typical values are between 5 and 50 [55]. The best perplexity found was 38 with an accuracy value of 96.83% reached by the combination of the *t*-SNE and *k*NN methods.

**Figure 8:**
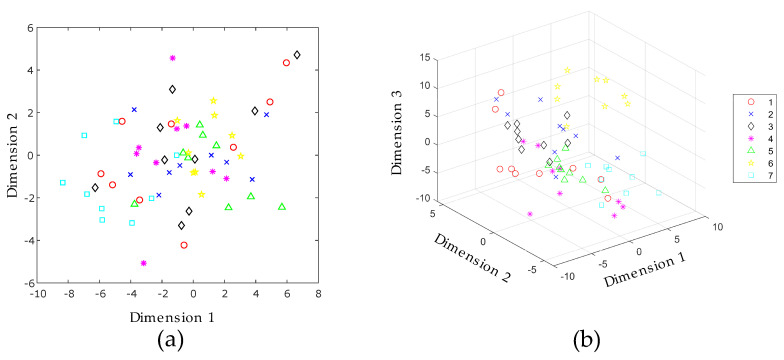
(**a**) Two-dimensional and (**b**) three-dimensional scatter plots after applying *t*-SNE for a perplexity of 38. The numbers in the legend refers to the seven classes of aqueous matrices as follows: red wine (1) white spirit (2), beer (3), black tea (4), oolong tea (5), maofeng tea (6), and pu′er tea (7).

Figure 8 shows the two-dimensional and three-dimensional scatter plots obtained with the *t*-SNE algorithm. In these diagrams it is observed that the classes cannot be easily distinguished, however, the special emphasis is made on the *t*-SNE method, as it had the best behavior in the classification results obtained with *k*NN and leave one out cross validation.

**Figure 9:**
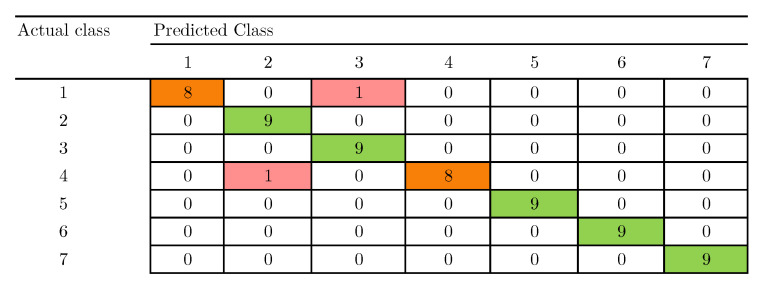
Confusion matrix obtained after applying *t*-SNE and 2-NN with LOOCV.

The confusion matrix presented in Figure 9 was obtained after applying the *t*-SNE algorithm combined with the *k*NN classification model. This confusion matrix represents an accuracy of 96.83%. To the best of our knowledge, this paper presents, for the first time, an accuracy of 96.83% in the seven aqueous matrices dataset, which surpasses the aforementioned results of the article developed by Liu et al. [13] (84.13%) and the work by Liu et al. in 2020 [12] (95.24%). It should be noted that the differences in accuracy between the works by Liu et al., and the methodology developed in this work are due to the fact that each paper uses different methods of feature extraction, this comparison can be observed in the Table 3. It can be observed in the confusion matrix of Figure 9 that the classes of white spirit (2), beer (3), oolong tea (5), maofeng tea (6), and pu′er tea (7) are totally classified, while those of red wine (1) and black tea (4) present a sample error per class.

**Figure 10:**
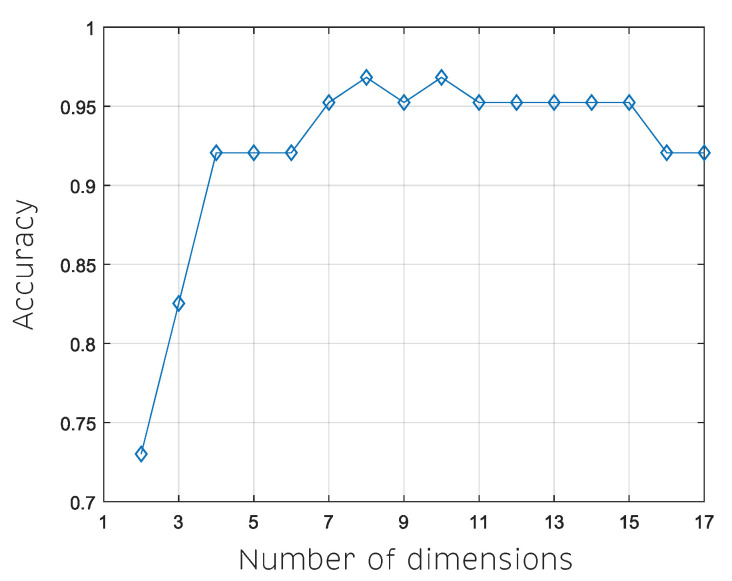
Variation of the number of target dimensions obtained by the *t*-SNE algorithm as input of the *k*NN classification model.

A variation of the number of target dimensions was obtained by the *t*-SNE algorithm as input to the *k*NN classification model. The best classification accuracy of 96.83% was obtained when 8 and 10 dimensions were selected as targets in the dimensionality reduction algorithm. As shown in Figure 10, when the number of dimensions increases, the accuracy also increases. However, after a determined number of dimensions, the accuracy behavior stabilizes.

Figure 10 shows the accuracy behavior when varying the number of target dimensions of the *t*-SNE algorithm. Here, the number of dimensions is seen as an important tuning parameter. Thus, we inspect the behavior of accuracy between 2 and 17 dimensions. We found that the best dimensions to embed the data with the *t*-SNE algorithm are the eight and 10 dimensions. This shows the possibility of having not only one but more optimal dimensions in the dimensionality reduction process. The reason is that, in each different dimension, the distances between classes are different. The *t*-SNE algorithm maximizes the between-classes separability and within-class compactness. In this case, in the search for the optimal dimensions, the KNN algorithm with two neighbors as classifier is set and fixed. Finally, different STD were obtained, for example, in the case of eight dimensions, 0.9683±0.018 was obtained and 0.9683±0.011 was obtained for 10 dimensions. Therefore, the best accuracy was finally obtained after performing a tuning process. First, the perplexity parameter in *t*-SNE algorithm was defined as 38. Second, it was found that the accuracy of 96.83% was reached by the eight and 10 dimensions, which shows that the accuracy depends on the selected target dimension. This occurs because the KNN classifier algorithm responds in the best way to the feature vectors created with the eight and 10 target dimensions.

Table 4 shows the classification accuracy results given by the LOOCV of the compared supervised machine learning classifiers varying the manifold learning method. The best combination of methods was *t*-SNE with *k*NN reaching an accuracy of 96.83%. From Table 4, it can be detected that the behavior of the manifold learning algorithms fluctuates with a similar trend, depending mainly on the classifier used. In general, it can be observed that the best accuracies were obtained by the KNN classifier. The next algorithms in terms of classification accuracy were SVM and MLP ANN. Finally, the classifying algorithms with the worst performance were Gaussian process classifier and Adaboost, respectively.

A comparative study was performed to show the accuracy behavior of each manifold learning algorithm when the number of target dimensions changes. Besides, the results with and without the application of the MCGS method at the beginning of data processing are shown in Table 5 and Table 6, respectively. The accuracy values presented in Table 4, Table 5 and Table 6 correspond to the average and standard deviation of classification accuracies, these values were obtained after performing *n* = 10 experiments. It can be observed that the application of the MCGS method generates an increase in the classification accuracy of all manifold learning algorithms.

As reported in Table 6, the size of the feature vector that enters to the *k*NN classifier varies from two to seventeen dimensions. In general, the worst behavior was obtained by the Laplacian Eigenmaps algorithm, while the best behavior was obtained by *t*-SNE algorithm. In Table 6, *t*-SNE was the only method capable of achieving an accuracy of 96.83% when 8 and 10 dimensions were used. When 4 dimensions were used Isomap reaches 95.24% and Laplacian Eigenmaps reached a maximum of 92.06% of classification accuracy. Furthermore, LLE reaches 92.06% when 12 dimensions were selected, while the maximum accuracy obtained for modified LLE was 92.06% when 5 dimensions were used. Finally, the best behavior for Hessian LLE with 90.48% and LTSA with 88.89% were reached for two dimensions.

## 6. Conclusions

The developed artificial taste recognition methodology allowed for correctly classifying aqueous matrices measured through a MLAPV electronic tongue. The leave-one-out cross validation was used, as the data set selected to validate the methodology had a few samples. The classes in the data set belong to 7 different aqueous matrices with 9 samples per class for a total of 63 samples. With the application of this artificial taste recognition methodology, the best results yielded 96.83% of classification accuracy, which means that it only mistook 2 out of the 63 total samples.

This methodology is composed of six stages: data unfolding, data normalization through MCGS, dimensionality reduction with a manifold learning algorithm, classification through a *k*NN machine learning model, cross validation, and performance measures calculation. In this study, seven manifold learning algorithms were compared: Isomap, Laplacian Eigenmaps, modified LLE, Hessian LLE, LTSA, LLE, and *t*-SNE. The findings indicate that the best algorithm was *t*-SNE, as it achieved the most accurate results.

The parameters of the algorithms influence the accuracy of classification and it is recommended to calibrate them. Particularly, in the *t*-SNE algorithm, the perplexity parameter exerted the most influence. Additionally, the variation of the number of neighbors in the *k*NN algorithm was examined, and k=2 and Euclidean distance were found to be the best selections. Moreover, the number of target dimensions obtained by the feature extraction algorithm influenced the results in the classification accuracy. In particular, the best classification accuracy results were achieved when the *t*-SNE algorithm reduced the dimensionality of the original data to 8 and 10 dimensions.

For future studies, the implementation of the developed methodology will be examined in other types of electronic tongues, such as those that are potentiometry- or cyclic voltammetry-based. The evaluation of other types of classifiers and the development of a portable instrument for measuring time and accuracy when implementing the methodology is also suggested.

## Figures and Tables

**Table 1:** Summary of different works related with pattern recognition methodologies in MLAPV electronic tongues.

ID	Reference	Electronic Tongue Type	Balanced/ Unbalanced	Number of Classes	Data Processing Stages	Best Combination of Methods	Cross Validation Method	Best Recognition Accuracy
1	[36]	MLAPV	Balanced	5	Normalization Feature Extraction Classifier	Normalization 0–1 STFT CNN-AFE	5 fold cross validation	99.9%
2	[1]	MLAPV	Unbalanced	13	Normalization Feature Extraction Classifier	Group Scaling PCA KNN	5 fold cross validation	94.74%
3	[14]	MLAPV	Unbalanced	13	Filter Feature Selection Feature Extraction Classifier	sliding window-based smooth filter LDPP KELM	5 fold cross validation	98.22%
4	[13]	MLAPV	Balanced	7	Feature Selection Classifier	ASF-DWT KNN	Leave one out cross validation	84.13%
5	[37]	LAPV	Balanced	4	Feature Selection Classifier	DWT ELM	Hold out cross validation	95%
6	[12]	MLAPV	Balanced	7	Feature Extraction Classifier	FSE KELM	Leave one out cross validation	95.24%
7	[38]	MLAPV	Balanced	5	Normalization Feature Selection Classifier	Baseline substraction +autoscale DWT RBF ANN	10 fold cross validation	98.33%

**Table 2:** Data set of 7 aqueous matrices used to validate the developed artificial taste recognition methodology.

ID	Aqueous Matrices	Samples
1	red wine	9
2	white spirit	9
3	beer	9
4	black tea	9
5	oolong tea	9
6	maofeng tea	9
7	pu’er tea	9

**Table 3:** Comparison of achieved classification accuracy in the seven aqueous matrices multifrequency large amplitude pulse voltammetry (MLAPV) electronic tongue dataset.

Research Articles	Methods	Accuracy
Liu et al., 2018 [13]	ASF-DWT + KNN	84.13%
Liu et al., 2020 [12]	FSE + KELM	95.24%
In the present article	MCGS + *t*-SNE + KNN	96.83%

**Table 4:** Accuracy average and standard deviation of classification accuracies, values obtained after performing *n* = 10 experiments. Varying the classifiers with respect to the manifold learning algorithms. The number of target dimensions was equal to 8.

Classifier	*t*-SNE	Isomap	Laplacian	LLE	Modified LLE	Hessian LLE	LTSA
KNN	0.9683 ± 0.018	0.8730 ± 0.028	0.8413 ± 0.038	0.8730 ± 0.017	0.7778 ± 0.022	0.7619 ± 0.027	0.8254 ± 0.022
SVM	0.7940 ± 0.034	0.7940 ± 0.032	0.7780 ± 0.038	0.7460 ± 0.035	0.7300 ± 0.003	0.6670 ± 0.014	0.6980 ± 0.024
MLP ANN	0.7619 ± 0.022	0.7777 ± 0.012	0.7460 ± 0.029	0.3968 ± 0.025	0.3968 ± 0.005	0.3492 ± 0.019	0.3968 ± 0.014
Adaboost	0.2857 ± 0.016	0.2857 ± 0.005	0.2698 ± 0.022	0.2857 ± 0.019	0.1428 ± 0.012	0.2698 ± 0.022	0.1269 ± 0.003
Gaussian Process	0.4444 ± 0.005	0.4285 ± 0.027	0.5396 ± 0.024	0.6031 ± 0.015	0.5873 ± 0.017	0.4920 ± 0.027	0.6666 ± 0.005

**Table 5:** Accuracy Average and standard deviation of classification accuracies, values obtained after performing *n* = 10 experiments. varying the dimensions with respect to the manifold learning algorithms. Data without MCGS applied.

D	*t*-SNE	Isomap	Laplacian	LLE	Modified LLE	Hessian LLE	LTSA
2	0.7460 ± 0.045	0.7143 ± 0.003	0.5873 ± 0.005	0.7460 ± 0.011	0.6825 ± 0.003	0.6667 ± 0.023	0.7302 ± 0.007
3	0.7937 ± 0.034	0.8095 ± 0.026	0.7937 ± 0.011	0.7619 ± 0.023	0.7302 ± 0.023	0.7619 ± 0.006	0.7778 ± 0.011
4	0.8730 ± 0.017	0.9048 ± 0.033	0.8413 ± 0.003	0.7460 ± 0.010	0.8413 ± 0.008	0.8571 ± 0.008	0.7778 ± 0.017
5	0.8889 ± 0.028	0.8571 ± 0.005	0.8571 ± 0.034	0.7937 ± 0.005	0.8413 ± 0.017	0.8571 ± 0.003	0.8254 ± 0.020
6	0.9365 ± 0.022	0.8730 ± 0.011	0.8889 ± 0.025	0.8413 ± 0.003	0.8095 ± 0.003	0.8571 ± 0.011	0.8095 ± 0.005
7	0.8889 ± 0.013	0.8571 ± 0.028	0.8413 ± 0.028	0.7937 ± 0.033	0.8413 ± 0.037	0.8095 ± 0.033	0.8095 ± 0.016
8	0.9206 ± 0.015	0.8413 ± 0.022	0.8254 ± 0.017	0.8571 ± 0.022	0.7619 ± 0.005	0.7937 ± 0.024	0.6825 ± 0.003
9	0.9206 ± 0.016	0.8254 ± 0.027	0.7619 ± 0.003	0.8730 ± 0.019	0.7460 ± 0.011	0.7460 ± 0.020	0.6667 ± 0.008
10	0.9048 ± 0.003	0.8254 ± 0.011	0.7143 ± 0.011	0.8413 ± 0.004	0.6825 ± 0.018	0.7460 ± 0.027	0.7460 ± 0.005
11	0.9206 ± 0.034	0.8254 ± 0.009	0.7937 ± 0.019	0.8730 ± 0.008	0.5873 ± 0.017	0.7460 ± 0.029	0.6825 ± 0.014
12	0.9206 ± 0.005	0.8889 ± 0.003	0.7937 ± 0.018	0.8571 ± 0.005	0.6190 ± 0.009	0.6508 ± 0.015	0.7460 ± 0.016
13	0.9048 ± 0.018	0.8413 ± 0.012	0.6825 ± 0.008	0.7778 ± 0.015	0.6190 ± 0.003	0.7460 ± 0.012	0.6667 ± 0.023
14	0.9206 ± 0.008	0.8095 ± 0.017	0.7302 ± 0.004	0.8095 ± 0.019	0.6825 ± 0.030	0.6508 ± 0.016	0.6825 ± 0.027
15	0.9048 ± 0.025	0.8413 ± 0.013	0.6667 ± 0.009	0.7937 ± 0.023	0.6349 ± 0.032	0.6508 ± 0.005	0.6825 ± 0.004
16	0.8571 ± 0.003	0.8413 ± 0.007	0.6825 ± 0.029	0.7937 ± 0.027	0.6508 ± 0.011	0.6825 ± 0.033	0.7460 ± 0.026
17	0.8730 ± 0.025	0.8413 ± 0.015	0.6825 ± 0.005	0.7937 ± 0.035	0.6508 ± 0.012	0.6825 ± 0.026	0.6667 ± 0.033

**Table 6:** Accuracy varying the dimensions with respect to the manifold learning algorithms. Data with MCGS applied.

D	*t*-SNE	Isomap	Laplacian	LLE	Modified LLE	Hessian LLE	LTSA
2	0.7302 ± 0.039	0.8730 ± 0.025	0.8413 ± 0.008	0.7937 ± 0.007	0.8571 ± 0.011	0.9048 ± 0.003	0.8889 ± 0.032
3	0.8254 ± 0.013	0.9048 ± 0.038	0.8254 ± 0.005	0.8254 ± 0.005	0.8095 ± 0.027	0.8730 ± 0.032	0.8571 ± 0.011
4	0.9206 ± 0.025	0.9524 ± 0.011	0.9206 ± 0.012	0.8889 ± 0.009	0.9048 ± 0.008	0.8889 ± 0.022	0.8571 ± 0.006
5	0.9206 ± 0.025	0.8889 ± 0.007	0.9048 ± 0.017	0.8571 ± 0.011	0.9206 ± 0.009	0.8730 ± 0.029	0.8730 ± 0.005
6	0.9206 ± 0.047	0.8889 ± 0.000	0.8889 ± 0.004	0.8571 ± 0.005	0.8730 ± 0.013	0.8730 ± 0.005	0.8889 ± 0.016
7	0.9524 ± 0.017	0.8889 ± 0.010	0.8730 ± 0.027	0.8730 ± 0.021	0.8254 ± 0.018	0.8254 ± 0.008	0.8889 ± 0.025
8	0.9683 ± 0.018	0.8730 ± 0.028	0.8413 ± 0.038	0.8730 ± 0.017	0.7778 ± 0.022	0.7619 ± 0.027	0.8254 ± 0.022
9	0.9524 ± 0.015	0.8730 ± 0.033	0.8730 ± 0.026	0.8889 ± 0.005	0.7143 ± 0.014	0.7937 ± 0.035	0.7937 ± 0.034
10	0.9683 ± 0.011	0.8730 ± 0.040	0.8571 ± 0.029	0.8889 ± 0.020	0.7619 ± 0.005	0.7937 ± 0.012	0.7937 ± 0.003
11	0.9524 ± 0.019	0.9206 ± 0.032	0.7937 ± 0.022	0.8889 ± 0.022	0.6508 ± 0.027	0.7619 ± 0.014	0.8413 ± 0.016
12	0.9524 ± 0.011	0.9206 ± 0.012	0.8095 ± 0.020	0.9206 ± 0.029	0.6508 ± 0.010	0.7619 ± 0.022	0.8254 ± 0.018
13	0.9524 ± 0.011	0.9206 ± 0.037	0.7619 ± 0.014	0.8889 ± 0.032	0.6349 ± 0.032	0.7460 ± 0.029	0.7937 ± 0.015
14	0.9524 ± 0.015	0.9048 ± 0.022	0.7460 ± 0.015	0.9048 ± 0.022	0.6349 ± 0.027	0.7937 ± 0.009	0.8413 ± 0.003
15	0.9524 ± 0.015	0.8413 ± 0.024	0.7619 ± 0.018	0.8571 ± 0.008	0.6190 ± 0.003	0.7460 ± 0.007	0.7937 ± 0.005
16	0.9206 ± 0.014	0.8413 ± 0.013	0.7778 ± 0.014	0.8730 ± 0.021	0.6508 ± 0.005	0.8254 ± 0.011	0.7937 ± 0.034
17	0.9206 ± 0.014	0.8413 ± 0.015	0.6825 ± 0.010	0.8889 ± 0.026	0.6508 ± 0.018	0.8254 ± 0.023	0.7937 ± 0.023

## Abbreviations

The following abbreviations are used in this manuscript:

ASF	active feature selection
DWT	discrete wavelet transform
FN	false negative
FP	false positive
*k*NN	*k* nearest neighbors
LDA	linear discriminant analysis
LDPP	local discriminant preservation projection
LLE	locally linear embedding
LOOCV	leave-one-out cross validation
LTSA	local tangent space alingment
MCGS	mean-centered group scaling
MLAPV	multifrequency large amplitude pulse signal voltammetry
PCA	principal component analysis
RBF	radial basis function
SHM	structural health monitoring
TN	true negative
TP	true positive
*t*-SNE	*t*-distributed stochastic neighbor embedding
